# Infected Solitary Renal Cyst in an Elderly Woman With Chronic Urinary Retention

**DOI:** 10.7759/cureus.100795

**Published:** 2026-01-05

**Authors:** Yusuke Ito, Koichiro Sunada

**Affiliations:** 1 Department of Family Practice, Azusawa Hospital, Itabashi, JPN

**Keywords:** chronic urinary retention, computed tomography (ct), infected renal cyst, renal cyst, spinal canal stenosis, ultrasonography (us)

## Abstract

Solitary renal cysts are common in elderly individuals and are usually asymptomatic. Cyst infection is rare but should be recognized as a potential complication. We report an 89-year-old woman with an infected solitary renal cyst who presented with atypical symptoms, including only dizziness, anorexia, and malaise, without localizing signs. Laboratory tests showed elevated inflammatory markers and pyuria, and *Escherichia coli* was isolated from urine. Imaging revealed enlargement and wall thickening of a Bosniak category II right renal cyst with debris-like echoes. She improved with antibiotics alone. Chronic urinary retention due to neurogenic bladder was considered a possible contributing factor to the cyst infection.

## Introduction

Solitary renal cysts are the most common acquired kidney lesions and are often detected incidentally with widespread use of ultrasonography and computed tomography (CT) [1]. They are thought to arise from weakening of the tubular basement membrane in the distal convoluted or collecting ducts, leading to diverticulum formation and subsequent cyst development [2]. Age-related fragility of the tubular basement membrane has been demonstrated, making aging a major risk factor for the development of renal cysts [2,3]. The prevalence increases with age, estimated to be approximately 20% by the age of 40 years and over 30% by the age of 60 years [1,2]. Autopsy studies have shown that nearly half of individuals aged over 50 years have one or more renal cysts [1,2].

Solitary renal cysts are usually asymptomatic but may cause symptoms due to enlargement or complications such as hemorrhage, rupture, or infection [2]. Cyst infection is common in autosomal dominant polycystic kidney disease (ADPKD) but rare in solitary renal cysts; a retrospective review of 200 cysts found infection in only 2.5% of cases [2,4].

Infected solitary renal cysts generally present with symptoms suggestive of acute infection, such as fever, flank or abdominal pain, or costovertebral angle tenderness, which can serve as diagnostic clues [5,6]. We report a case of an elderly patient who presented only with nonspecific symptoms, that is, dizziness, anorexia, and general malaise, which complicated the diagnosis.

## Case presentation

**Figure 1:**
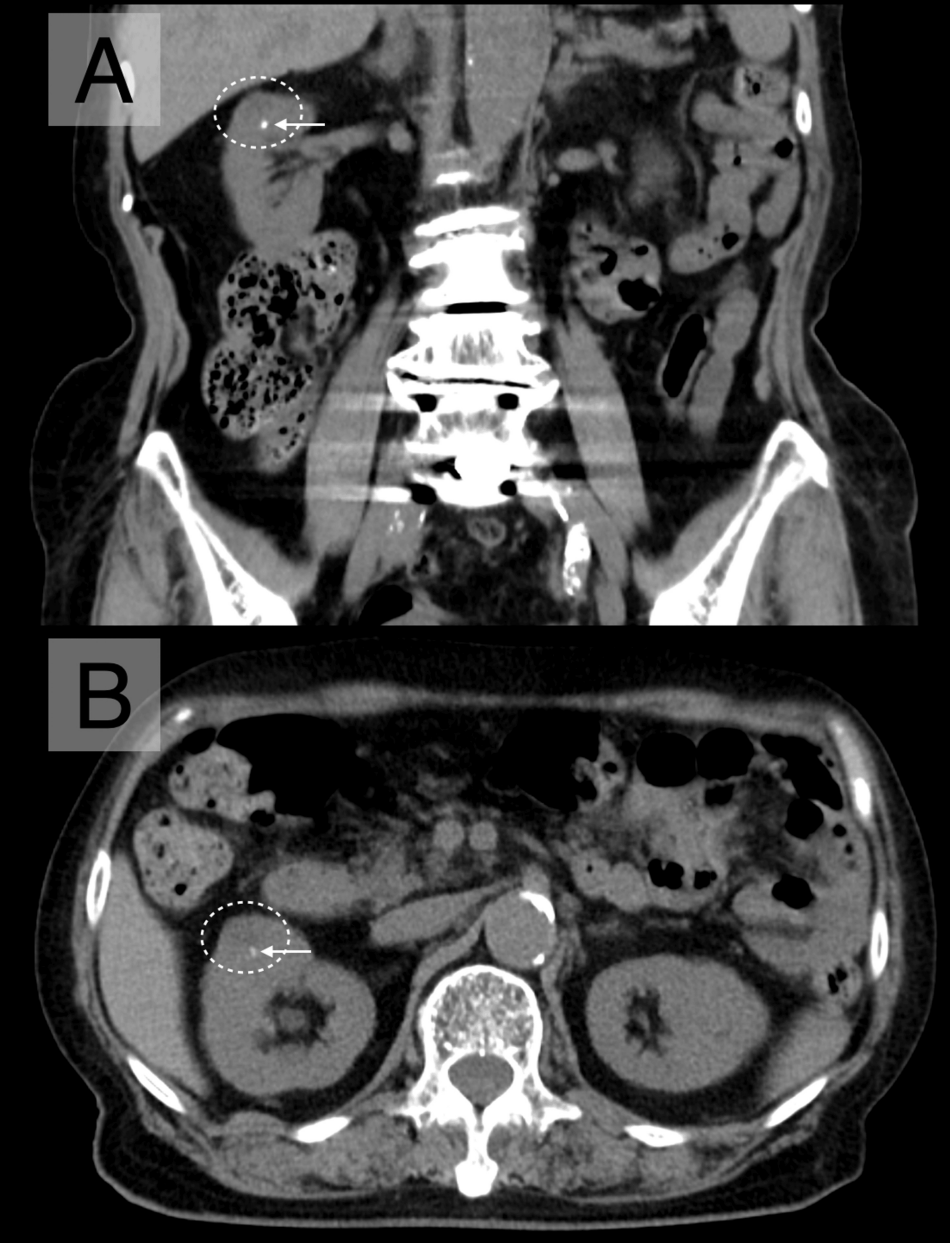
Abdominal CT six months before admission. An abdominal CT performed six months before admission showed a 10-mm solitary right renal cyst (dotted circles) with fine wall calcification (arrow), classified as Bosniak category II. (A) Coronal and (B) axial views.

An 89-year-old woman was admitted with vague symptoms, including dizziness, anorexia, and general malaise. She had a history of chronic respiratory failure, managed with home oxygen therapy at 1 L/min. Ten years earlier, she had undergone posterior lumbar decompression and fusion for lumbar spinal stenosis; however, persistent back pain and lower limb numbness remained, for which she was receiving regular sacral epidural block therapy. Six months earlier, abdominal CT had incidentally revealed a 10-mm solitary right renal cyst with fine wall calcification, classified as Bosniak category II (Figure 1) [1]. No history of trauma was reported after its identification.

On admission, her vital signs were as follows: blood pressure 116/86 mmHg, pulse 76 bpm, temperature 36.2°C, and oxygen saturation 95% with 1 L/min oxygen. Physical examination revealed no back or abdominal tenderness; however, a detailed history revealed that she had experienced intermittent epigastric discomfort and nausea during the past month. Laboratory tests showed elevated C-reactive protein (8.55 mg/dL; reference range, 0-0.30 mg/dL) and leukocytosis (11550/μL; reference range, 3500-9000/μL) (Table 1). Mild renal dysfunction was also noted, with a serum creatinine level of 1.56 mg/dL (reference range, 0.45-0.80 mg/dL), and improved after adequate hydration. Urinalysis revealed pyuria (30-49/HPF), and urine culture grew *Escherichia coli*.

**Table 1:** Laboratory findings on admission.

Parameter	Result	Reference range
White cell count (/μL)	11550	3500-9000
Neutrophils (%)	78.1	40-70
Lymphocytes (%)	14.5	20-50
Monocytes (%)	6.0	3-11
Eosinophils (%)	1.1	0-5
Basophils (%)	0.3	0-2
Red blood cell (10^4^/μL)	368	427-570
Hemoglobin (g/dL)	11.1	13.5-17.6
Platelet count (10^4^/μL)	42.2	13-37
Total protein (g/dL)	8.3	6.7-8.3
Albumin (g/dL)	3.0	3.8-5.2
Blood urea nitrogen (mg/dL)	34.0	8-20
Creatinine (mg/dL)	1.56	0.6-1.1
Sodium (mEq/L)	136	135-148
Potassium (mEq/L)	5.2	3.5-5
Chloride (mEq/L)	104	98-110
Asparate aminotransferase (U/L)	6	10-40
Alanine aminotransferase (U/L)	3	5-42
Lactate dehydrogenase (U/L)	165	124-222
Creatine kinase (U/L)	18	40-200
C-reactive protein (mg/dL)	8.55	0-0.3
Glucose (mg/dL)	123	70-109
Hemoglobin A1c (%)	6.4	4.6-6.2

**Figure 2:**
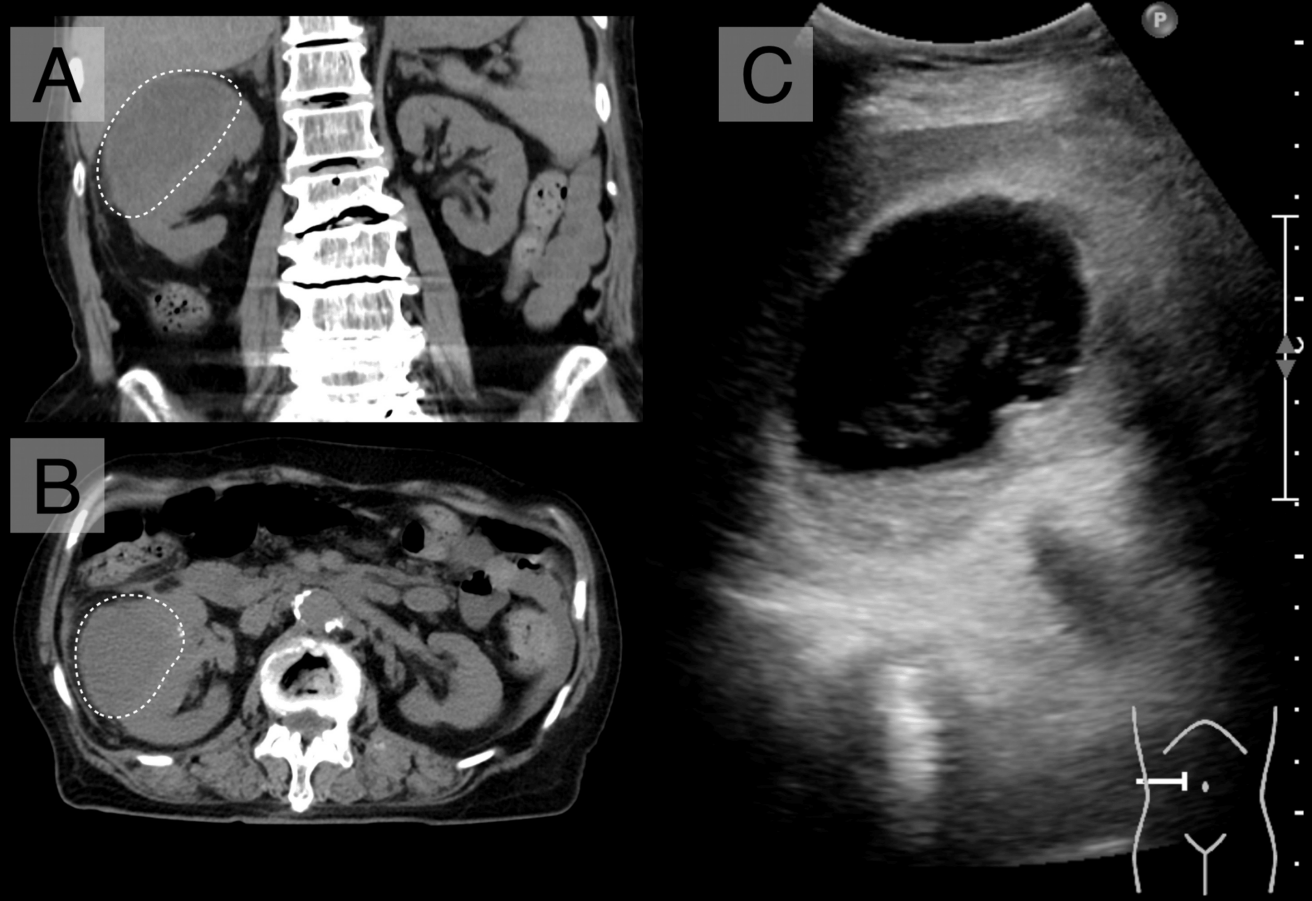
Imaging findings on admission. A non-contrast CT showed an enlarged right renal cyst (75 × 55 × 38 mm, dotted circles) with increased density and wall thickening accompanied by pericystic fat stranding. (A) Coronal and (B) axial views. (C) Abdominal ultrasonography showing debris-like internal echoes.

A non-contrast CT demonstrated enlargement of the right renal cyst (75 × 55 × 38 mm), increased density, and wall thickening with pericystic fat stranding (Figure 2A, 2B), and debris-like internal echoes were observed on abdominal ultrasonography (Figure 2C), consistent with an infected solitary renal cyst. Bladder ultrasonography revealed impaired bladder emptying, with a bladder volume of 190 mL before voiding and 150 mL after voiding, without hydronephrosis. Further history revealed painless, longstanding voiding difficulty, suggesting chronic urinary retention. In the absence of hydronephrosis and renal dysfunction, low-pressure chronic retention was suspected [7], and conservative therapy with urapidil (30 mg/day) was initiated.

On day 3 of hospitalization, intravenous ceftriaxone 2 g/day was started and later switched to oral levofloxacin 250 mg/day. The total course of antibiotics lasted 18 days, resulting in improvement of inflammatory markers and symptoms. Given the moderately large cyst, a urological consultation was requested; however, drainage was not performed in view of her advanced age and favorable clinical course.

**Figure 3:**
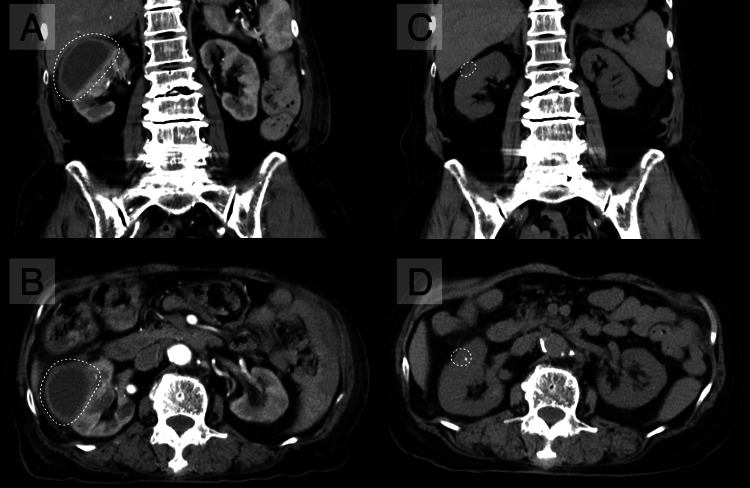
Follow-up imaging after treatment. A follow-up contrast-enhanced CT on day 34 showed a reduction in cyst size (67 × 50 × 33 mm, dotted circles), with no septa or enhancing soft-tissue components. (A) Coronal and (B) axial views. A follow-up non-contrast CT performed five months after discharge showed improvement of the enlarged cyst (dotted circles). (C) Coronal and (D) axial views.

On day 34, a follow-up contrast-enhanced CT showed a reduction in the cyst size (67 × 50 × 33 mm). The fine wall calcification remained stable, and no septa or enhancing soft-tissue components adjacent to the wall were observed, ruling out cystic neoplasm (Figure 3A, 3B). Despite conservative therapy with urapidil, follow-up bladder ultrasonography showed 700 mL of urinary retention without any sensation of voiding, consistent with a neurogenic bladder. Clean intermittent self-catheterization was considered but declined by the patient; therefore, long-term indwelling catheterization was initiated after consultation with urology. She was discharged on day 43. A follow-up non-contrast CT performed five months after discharge showed improvement of the enlarged cyst (Figure 3C, 3D).

## Discussion

We report a case of an elderly woman with an infected solitary renal cyst presenting atypically, likely secondary to an ascending urinary tract infection associated with chronic urinary retention.

Solitary renal cysts are widely evaluated using the Bosniak renal cyst classification system, which is based on imaging appearance and enhancement (categories I, II, IIF, III, and IV) [1]. This classification is widely applied for the diagnosis and management of cystic renal masses, as well as for estimating the risk of malignant transformation [1]. According to Bosniak classification, categories I and II are benign, and only 2-4% of cases become symptomatic due to enlargement or complications such as hemorrhage, infection, or rupture [2]. Therefore, a cyst classified as Bosniak category II, as in the present case, typically does not raise clinical concern.

In the present case, the following points provide clinically important insights. First, chronic urinary retention can lead to infection of a solitary renal cyst. Second, the patient’s atypical symptoms complicated the diagnosis, and imaging was essential for establishing the diagnosis. Third, despite the moderately large size of the infected cyst, the infection was successfully managed with conservative treatment.

Renal cyst infection is hypothesized to arise either from ascending urinary tract infection or from hematogenous spread [8]. In ADPKD, cyst infection is common in both sexes, and negative urine cultures with evidence of hematogenous spread are relatively frequent [9]. One hypothesis is that this reflects the high prevalence of dialysis in patients with ADPKD, who are often completely anuric, making urinary tract infection less likely [9]. In contrast, most cases of infected solitary renal cysts involve women (92%), often accompanied by pyuria [5,6]. In general, women are more prone to urinary tract infections, which supports the view that most cases result from ascending infection [5]. In the present case, the detection of pyuria suggested that the cyst infection was secondary to a urinary tract infection.

Urinary retention has historically been classified as acute or chronic. Acute urinary retention is defined as a sudden-onset condition that is often painful and requires intervention to relieve symptoms, whereas chronic urinary retention may be asymptomatic or preceded by low-volume voiding, increased frequency, or difficulty initiating and maintaining urination [10]. The main causes of urinary retention are obstructive, with other causes including infectious and inflammatory, iatrogenic, or neurologic causes [11]. Among patients requiring long-term urinary catheterization, obstructive causes due to benign prostatic hypertrophy are common in men, whereas neurologic causes are more frequent in women [12]. In the present case, a history of spinal stenosis may have contributed to chronic urinary retention as a neurologic cause. Previous studies have reported that voiding dysfunction can persist even after surgical treatment of spinal stenosis [13-15]. In addition, age-related neurologic, anatomic, and biochemical changes in the bladder and lower urinary tract may have contributed [14,16]. Therefore, advanced age and a history of spinal stenosis, even after surgery, are risk factors for chronic urinary retention and its complications, including renal cyst infection.

Definitive diagnosis of infected renal cysts generally relies on cyst aspiration and culture, which is considered the gold standard and requires a cyst aspirate containing both white blood cells and pathogens [17]. However, cyst aspiration can often be challenging due to advanced age, poor general condition, or technical and institutional limitations, and the diagnosis is frequently made empirically [17]. In ADPKD, diagnostic criteria based on clinical symptoms, laboratory findings, and CT or MRI findings without cyst aspiration have been proposed, and noninvasive diagnostic approaches are also important for infected solitary renal cysts [9].

The characteristic symptoms of infected solitary renal cysts are fever (53%), flank pain (50%), abdominal pain (42%), abdominal tenderness (39%), costovertebral angle tenderness (35%), and a palpable mass (23%) [6]. These findings usually indicate acute infection and serve as important diagnostic clues. The younger mean age of patients in that review (27 years) may partly explain differences in clinical presentation compared with the present case [6]. Other reports indicate that not only fever but also marked inflammatory findings strongly suggest cyst infection: white blood cell exceeding 10,000/µL, C-reactive protein greater than 15 mg/dL, and temperature above 38°C [9]. As urinary tract infections in elderly patients are known to present atypically, infected solitary renal cysts in this population may also manifest atypically [18]. Therefore, even in the absence of typical symptoms, unexplained inflammatory findings in elderly patients should prompt further evaluation for a possible infectious source.

Imaging is useful for diagnosing cyst infection; however, cyst infection and cyst hemorrhage can show similar findings (such as increased density on CT and high intensity on MRI), making differentiation challenging [19]. Imaging features of cyst infection include fluid-fluid levels, wall thickening, pericystic fat stranding, and gas formation, in addition to increased CT density (cutoff value, 10.0 HU) and high intensity on T1-weighted (cutoff value, 1.0) and diffusion-weighted imaging (cutoff value, 4.0) [19]. Positron emission tomography has also been reported as a useful imaging modality for the diagnosis of cyst infection [19]. Characteristic ultrasound findings of infected renal cysts include minute debris, amorphous solid conglomerates, and internal septations [20]. In the present case, key findings were the debris sign on ultrasound, as well as increased density and wall thickening with pericystic fat stranding on CT.

Most treatment guidelines for infected renal cysts are based on patients with ADPKD, and the management of infected solitary renal cysts has not been established. Initial treatment generally involves antibiotics. Lipid-soluble antibiotics, particularly fluoroquinolone-based regimens, have been recommended because of their superior cyst penetration [8]. However, intravenous administration of water-soluble antibiotics, including penicillin- and cephalosporin-based agents, has been suggested to be effective for mild cases of cyst infection [9]. In the present case, the patient improved with water-soluble antibiotics, ceftriaxone, during the initial treatment.

Drainage is generally considered for cyst infections unresponsive to initial appropriate antibiotics or for large cysts [9]. Reported indications include antibiotic resistance, cysts larger than 5 cm, severe illness such as sepsis or disseminated intravascular coagulation, and recurrent infections [9]. Although the complication rate of drainage is low (2.75%), serious adverse events may occur; therefore, the procedure should be undertaken with caution [9]. Nephrectomy may be an option for refractory infection when drainage is technically unfeasible [9]. In the present case, despite a moderately enlarged infected solitary renal cyst (>5 cm), the patient responded well to initial antibiotic therapy, avoiding drainage. Given the procedural risks, antibiotic therapy alone may be a reasonable option in elderly patients without severe illness or risk factors (e.g., renal impairment, urolithiasis, or post-renal obstruction).

## Conclusions

This case highlights a rare complication of a solitary renal cyst (cyst infection possibly associated with chronic urinary retention) presenting with atypical symptoms. Despite being a rare complication, both urinary tract infections and solitary renal cysts are common in elderly patients, and clinicians should be aware of this potential association.
